# Multiresidue Method for Quantification of Sulfonamides and Trimethoprim in Tilapia Fillet by Liquid Chromatography Coupled to Quadrupole Time-of-Flight Mass Spectrometry Using QuEChERS for Sample Preparation

**DOI:** 10.1155/2018/4506754

**Published:** 2018-03-01

**Authors:** Kátia S. D. Nunes, Márcia R. Assalin, José H. Vallim, Claudio M. Jonsson, Sonia C. N. Queiroz, Felix G. R. Reyes

**Affiliations:** ^1^Department of Food Science, School of Food Engineering, University of Campinas, Rua Monteiro Lobato 80, 13083-862 Campinas, SP, Brazil; ^2^Embrapa Meio Ambiente, P.O. Box 69, 13820-000 Jaguariúna, SP, Brazil

## Abstract

A multiresidue method for detecting and quantifying sulfonamides (sulfapyridine, sulfamerazine, sulfathiazole, sulfamethazine, sulfadimethoxine, sulfamethoxazole, and sulfamethoxypyridazine) and trimethoprim in tilapia fillet (*Oreochromis niloticus*) using liquid chromatography coupled to mass spectrometry was developed and validated. The sample preparation was optimized using the QuEChERS approach. The chromatographic separation was performed using a C18 column and 0.1% formic acid in water and acetonitrile as the mobile phase in the isocratic elution mode. Method validation was performed based on the Commission Decision 2002/657/EC and Brazilian guideline. The validation parameters evaluated were linearity (*r* ≥ 0.99); limits of detection (LOD) and quantification (LOQ), 1 ng·g^−1^ and 5 ng·g^−1^, respectively; intraday and interdays precision (CV lower than 19.4%). The decision limit (CC*α* 102.6–120.0 ng·g^−1^ and 70 ng·g^−1^ for sulfonamides and trimethoprim, respectively) and detection capability (CC*β* 111.7–140.1 ng·g^−1^ and 89.9 ng·g^−1^ for sulfonamides and trimethoprim, respectively) were determined. Analyses of tilapia fillet samples from fish exposed to sulfamethazine through feed (incurred samples) were conducted in order to evaluate the method. This new method was demonstrated to be fast, sensitive, and suitable for monitoring sulfonamides and trimethoprim in tilapia fillet in health surveillance programs, as well as to be used in pharmacokinetics and residue depletion studies.

## 1. Introduction

**Figure 1:**
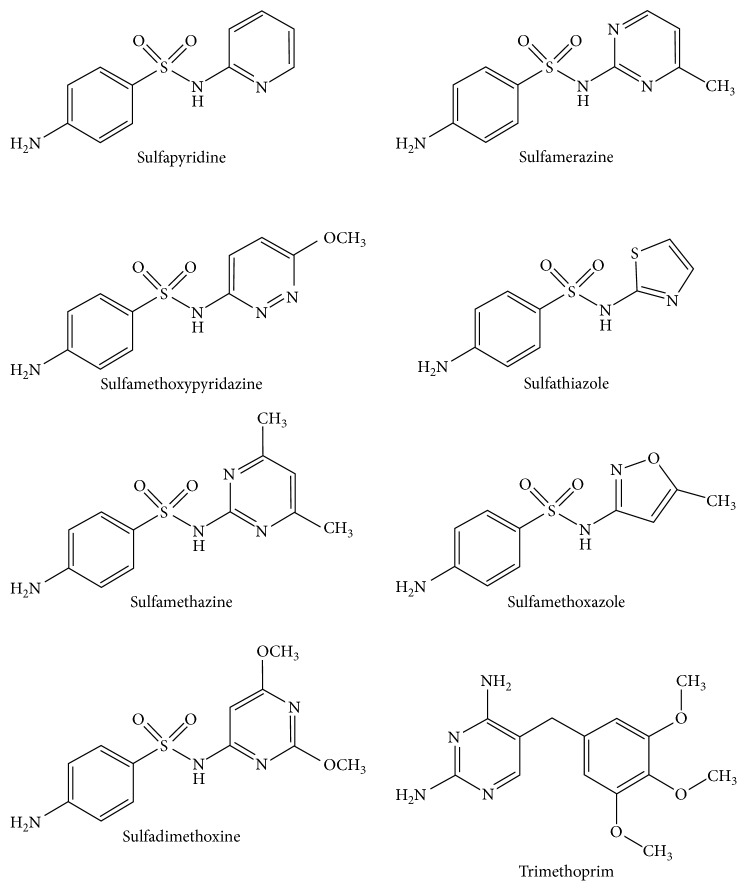
Chemical structures of the sulfonamides and trimethoprim.

Brazil is one of the five largest veterinary markets in the world, and aquaculture, in particular fish farming, is the fastest growing sector of animal food production in the country [1, 2]. In fish farming, antimicrobials, including sulfonamides, are used for the treatment of bacterial diseases. Sulfonamides (Figure 1) belong to an important group of synthetic antimicrobial agents that have been used in human and veterinary medicine for over 60 years. Recently, these drugs have been extensively employed in animals intended to produce food for human consumption since it is practically impossible to keep the production environment free of pathogenic organisms. Sulfonamides have become a useful tool for achieving high levels of productivity, thereby contributing to further growth, feed efficiency, and reduced mortality and morbidity [3]. However, sulfonamide residues are a major concern because of their potential risk to human health by development of bacterial resistance and adverse effects, such as allergic reactions, in hypersensitive people [4].

Trimethoprim (Figure 1) is a diaminopyrimidine antimicrobial agent, which is active against a wide range of Gram-positive and Gram-negative microorganisms including *Escherichia coli* and some *Klebsiella*, *Proteus*, and *Staphylococcus* species. In veterinary medicine, it is often used in combination with a sulfonamide to increase the antimicrobial activity of the sulfonamides but is excreted faster. Consequently, if no residues of sulfonamide are detectable, no residues of trimethoprim would be expected. Trimethoprim is of low acute mammalian toxicity, and there is no evidence for the potentiation of acute toxicity when it is administered in combination with a sulfonamide [5].

At its 40th session, the Codex Alimentarius Commission reported a maximum residue limit (MRL) value for sulfadimidine (sulfamethazine) of 100 *µ*g·kg^−1^ in muscle, for species not specified [6]. According to the European Commission Regulation (EU) No. 37/2010 [7], for the muscle of fin fish, the MRL value for individual sulfonamides, or the combined total residues of all substances belonging to the sulfonamide group, is 100 *µ*g·kg^−1^. In relation to trimethoprim, the MRL value is 50 *µ*g·kg^−1^. The MRL value relates to the muscle and skin in natural proportions. In Brazil, the use of sulfonamides in farm-raised fish is not permitted (it does not appear in the legislative framework) and, therefore, its use is considered out of label (prohibited substance). However, for monitoring purposes (and taking actions), the Brazilian National Plan for Control of Residues and Contaminants (PNCRC/Fish) establishes a reference limit of 100 *μ*g·kg^−1^ for the residue of the individual sulfonamides (sulfachlorpyridazine, sulfadoxine, sulfamerazine, sulfadiazine, sulfamethoxazole, sulfathiazole, sulfamethazine, sulfaquinoxaline, and sulfadimethoxine) or the sum of them. Trimethoprim is not considered under the PNCRC/Fish sampling plan [8].

Studies on the determination of antimicrobial residues in foods of animal origin began in Belgium, the Netherlands, and Luxembourg in the late 1960s and early 1970s. In most European countries, research on residues and their application in inspection of slaughtered animals started later [9]. In relation to the sample preparation step, strategies such as salting out liquid-liquid extraction [10], solid-liquid extraction [11], and microscale matrix solid-phase dispersion [12] have been employed to perform the extraction and cleanup of sulfonamides from fish and other biological matrices. More recently, Ziarrusta et al. [13] used focused ultrasound solid-liquid extraction (FUSLE) for extraction of fluoroquinolones from fish tissues. The FUSLE method improves the extraction yield of target analytes (organic compounds), quantitatively, from biota samples. Regarding the systems of separation and detection, the high performance liquid chromatography-tandem mass spectrometry (HPLC-MS/MS) is an analytical technique that has been used in the determination of veterinary drug residues. In this regard, a few sulfonamide multiresidue methods in food matrices have been described in the literature by this technique [14, 15]. For instance, Abdallah et al. [16] determined sulfonamide residues in sheep, pork, beef, chicken, and dromedary, Nebot et al. [17] in bovine milk, Tsai et al. [18] in different fish species, and Jansomboon et al. [19] in *Pangasius* catfish. Alternatively, a time-of-flight (TOF) mass spectrometer provides high sensitivity and accurate mass measurements (0.005 Da), enabling the detection of low concentrations (ng·g^−1^) of residues and contaminants in highly complex food matrices [15, 20]. Nevertheless, to our knowledge, there is no reported multiresidue method for the combined quantification of sulfonamides and trimethoprim in tilapia fillet using liquid chromatography coupled to quadrupole time-of-flight mass spectrometry (LC-QTOF/MS).

The aim of this study was to develop and validate a rapid, simple (without the need of solid-phase extraction (SPE) cartridges or similar materials), and reliable multiresidue method for the identification and quantification of sulfonamides and trimethoprim in tilapia fillets (*Oreochromis niloticus*) by LC-QTOF/MS, to be suitable for application in monitoring programmes as well as in pharmacokinetic and residue depletion studies. The sample preparation involved the QuEChERS (Quick, Easy, Cheap, Effective, Rugged, and Safe) approach as described by Lehotay et al. [21]. The validation was conducted in-house based on the Commission Decision 2002/657/EC [22] and Brazilian guideline [23]. To evaluate the precision of the method, analysis of tilapia fillet samples from fish exposed to sulfamethazine through feed (incurred samples) was also conducted.

## 2. Materials and Methods

### 2.1. Chemicals and Reagents

The sulfonamide analytical standards (sulfathiazole (STZ), sulfamethoxazole (SMX), sulfamerazine (SMR), sulfamethoxypyridazine (SMPD), sulfadimethoxine (SDMX), sulfapyridine (SP), sulfamethazine (SMZ)), and trimethoprim (TMP) were purchased from Sigma-Aldrich Company Ltd. (St. Louis, MO, USA). All analytical standards had a purity greater than 99.0%. Primary secondary amine (PSA) was obtained from United Chemical Technologies, Inc. (UCT Inc., Bristol, PA, USA), and formic acid (98%) was purchased from Sigma-Aldrich Company Ltd. (St. Louis, MO, USA). Anhydrous magnesium sulfate was supplied by J.T. Baker (Center Valley, PA, USA) and sodium acetate trihydrate from Spectrum Chemical Mfg., Corp. (New Brunswick, NJ, USA). Methanol (MeOH) and acetonitrile (ACN) were obtained from Honeywell Burdick & Jackson (Muskegon, MI, USA) and J.T. Baker (Center Valley, PA, US), respectively. All solvents were of HPLC grade, and all reagents were of analytical grade. Ultra-pure deionized water was obtained from a Milli-Q Plus water purification system (Millipore, Bedford, MA, USA). Filtration of the aqueous mobile phase was performed using polyvinylidene fluoride (PVDF) membranes, and polytetrafluoroethylene (PTFE) membranes were used for organic mobile-phase filtration, both with 0.22 *µ*m pore size obtained from Millipore (Bedford, MA, USA).

### 2.2. Instrumentation

The identification and quantitation of sulfonamides and trimethoprim was carried out using an UPLC-Q-TOF system comprising an Acquity UPLC system coupled to a hybrid quadrupole orthogonal time-of-flight (Q-TOF) mass spectrometer (SYNAPT HDMS Q-TOF mass spectrometer) with electrospray source ionization (ESI) in positive mode. The software of acquisition control and data treatment was the MassLynx version 4.1 (Waters Corp., Milford, MA, USA). For sample preparation, the following equipment were used: semianalytical balance (Tecnal; Boulder, CO, USA); analytical balance (Scientech, SA 210; Boulder, CO, USA); tubes stirring vortex type (IKA model MS1 Minishaker, 2700 rpm; Wilmington, DE, USA); refrigerated centrifuge (Thermo Scientific model Heraeus Multifuge 3 L-R; Madison, WI, USA); ultrasonic bath (Elma model Transsonic 660/H; Singen, Baden-Württemberg, Germany); Waring Commercial Blender, model 33BL79 (New Hartford, CT, USA); and Ultra-Turrax IKA, model TP 10N (Wilmington, DE, USA).

### 2.3. Solution Preparation

Standard stock solutions of SP, STZ, SMZ, SDMX, SMX, SMPD, SMR, and TMP were prepared in acetonitrile at 1000 *µ*g·mL^−1^, stored in 10 mL bottles, and kept at −20°C. These solutions were used for a maximum period of 1 month. The intermediate standard solutions were prepared daily by dilution of stock solutions in an appropriate buffer solution.

### 2.4. Blank and Incurred Fish Samples

The blank samples of tilapia (*Oreochromis niloticus*) with no detectable analyte concentration used for the development and validation of the analytical method were provided by a local producer (Rio Doce fish farm, São João da Boa Vista, SP) with a guarantee that the fish were not exposed to the compounds that were the analytical focus of this work. Nonetheless, to ensure the viability of the blank samples, they were analysed, and the chromatograms did not show the presence of any interference at the retention time corresponding to the studied analytes. For validation of the analytical method, blank samples and incurred samples (truly contaminated samples) were used, that is, samples of fish exposed to SMZ through feed, obtained from an experiment conducted at Embrapa Environment, Jaguariuna, SP, Brazil, where tilapia were given SMZ at a dose level of 422 mg·kg^−1^ body weight, for 11 consecutive days. The incurred samples used in this study were from fish slaughtered by thermal shock and immersion in an ice bath, 12 h after stopping medication. All samples were stored in a freezer (−20°C) until analysis [24]. The experiment with fish to obtain the incurred samples was approved by the Ethics Committee on Animal Experiments of Embrapa Environment (Protocol No. 001/2013) [25].

### 2.5. Sample Preparation by QuEChERS

**Figure 2:**
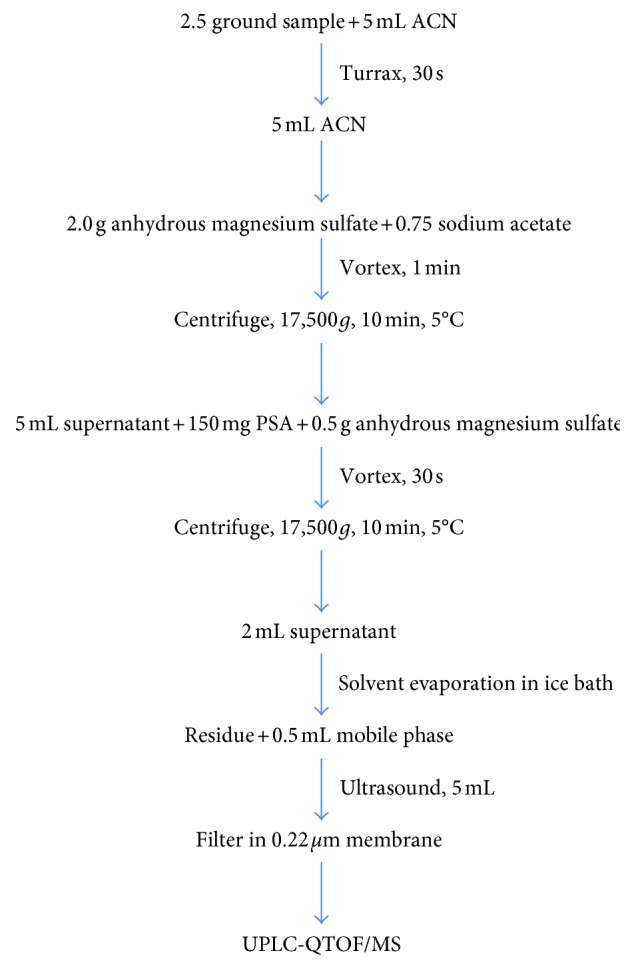
Schematic representation of the sample preparation procedure.

Tilapia fillet samples were ground using a domestic food processor. Triturated samples (2.5 g) were weighed in a 50 mL polypropylene tube, and ACN (5 mL) was added and then homogenized using a Turrax for 30 seconds. The homogenized sample was then added of 5 mL ACN, the tubes were shaken vigorously by vortexing for 1 min and placed in an ultrasonic bath for 5 min. Next, 2.0 g of anhydrous magnesium sulfate and 0.75 g of sodium acetate were added to the homogenized samples and vortexed for 1 min and centrifuged at 17,500 × g for 10 min, at 5°C. For sample cleanup, an aliquot of 5.0 mL of supernatant was volumetrically pipetted to another tube containing 150 mg of PSA and 0.5 g of anhydrous magnesium sulfate. The tube was subsequently vortexed for 30 seconds and centrifuged at 17,500 × g again for 5 min, at 5°C. A 2.0 mL aliquot of the supernatant was pipetted and transferred to another tube, and the solvent was completely evaporated under nitrogen stream, in an ice bath, to avoid losses of the analytes. Next, the residue was suspended in 0.5 mL of the mobile phase (ACN : 0.1% aqueous formic acid, 95 : 5 v/v). To facilitate the dissolution of analytes, the tubes were placed in ultrasonic bath for 5 min. Finally, the resulting extracts were filtered through a cellulose filter unit (0.22 *µ*m pore size) directly into the vial and injected in the LC-QTOF/MS system. A schematic representation of the sample preparation procedure is shown in Figure 2.

### 2.6. UPLC-QTOF/MS Conditions

The chromatographic separation was performed on a reversed-phase analytical column Poroshell EC-120 C18 (50 mm × 2.1 mm, 2.7 *µ*m), supplied by Agilent Technologies (Santa Clara, CA, USA) preceded by a similar precolumn (30 mm × 2.1 mm, 2.7 *µ*m). The chromatographic separation was performed at 25°C. The mobile phase was composed of (A) H_2_O : acetonitrile : formic acid (95 : 5 : 0.1%, v/v/v) and (B) H_2_O : acetonitrile : formic acid (5 : 95 : 0.1%, v/v/v), and the isocratic elution mode was used with 70% (A) and 30% (B). The flow rate was 0.2 mL·min^−1^ with a run time of 4 min and injection volume of 5 *µ*L.

The following ionization conditions were established for the ESI-QTOF/MS system: positive ionization mode, capillary voltage: 2.5 kV, detector voltage: 1.850 kV, sample cone voltage: 20.0 V, extraction cone voltage: 2.0 V, source temperature: 100°C, desolvation gas temperature: 300°C, nitrogen gas flow in the cone: 50 L·h^−1^, and desolvation flow: 400 L·h^−1^. The molecules of interest were quantified by monitoring the signal related to the protonated molecular ion *m/z* (M + H^+^). The sulfonamide and trimethoprim identity was confirmed by obtaining the accurate mass of the protonated molecular ion, as well as by the consideration of fragment ions in order to obtain the identification points (IPs) according to Commission Decision 2002/657/EC [22] (Table 1).

### 2.7. Validation Parameters

The purpose of this step was to establish the performance parameters and the minimum requirements of acceptance that must be satisfied such that the analytical method presented in this study is considered validated. The recommendations of the European Community [22] and the Guide to Analytical Methods Validation of the Brazilian Ministry of Agriculture, Livestock, and Supply [23] were used as reference to perform the method validation.

After optimization of the preparation procedure (extraction and cleanup), the validation of the analytical method was performed. The following validation parameters were evaluated: selectivity; linearity, sensitivity, and matrix effect; precision (intra- and interday); accuracy; and decision limit (CC*α*) and detection capability (CC*β*). The limit of detection (LOD) and limit of quantification (LOQ) were also assessed to evaluate the potential use of the analytical method in pharmacokinetic and residue depletion studies where lower LOD and LOQ are required. Selectivity of the method was evaluated by comparing the chromatograms obtained from blank samples (*n*=10) and the samples spiked with sulfonamides and trimethoprim standard solutions (*n*=10). The chromatograms were evaluated for the presence of the analytical signal at the same retention time observed for the mass-to-charge ratio (*m/z*) of the analytes of interest.

Linearity was established from analytical curves obtained by duplicate analysis of blank samples spiked with trimethoprim and sulfonamides in the following concentrations: 5.0, 12.5, 25.0, 50.0, 75.0, 100.0, 125.0, and 250.0 ng·g^−1^. The results were analysed by the method of least squares , and the linearity was expressed through the coefficient of determination (*R*^2^) which was adopted as *R*^2^ ≥ 0.99, as recommended by the Brazilian validation guide [23]. The matrix effect was evaluated by comparing three different concentrations (12.5, 50.0, and 100.0 ng·g^−1^) of sulfonamides and trimethoprim, prepared in solvent and fortified extracts. The evaluation was done by comparing the area of the analytical signal in solvent with the area of analyte in the fortified extracts. Accuracy was evaluated by recovery tests of the spiked blank matrix at three concentration levels (10.0, 20.0, and 40.0 ng·g^−1^) with five replicates of each spiked level, during 3 days. The results were expressed as mean values (*n*=15) of percentage of recoveries. The coefficient of variation (CV%) is also reported.

The precision of the method was determined in two steps: intraday precision (repeatability) and interdays precision (intermediate precision). Repeatability was expressed as the CV% of the results obtained with five replicates at three different concentrations (10.0, 20.0, and 40.0 ng·g^−1^) analysed on the same day by the same analyst. The intermediate precision was expressed by CV% of the results of three different concentrations with five replicates of each concentration on three different days by the same analyst.

The calculation of the decision limit (CC*α*) and the detection capability (CC*β*) was based on the Commission Decision 2002/657/EC [22]. The decision limit is defined as the lowest concentration level at which the method can discriminate with a statistical certainty of 1−*α* if the analyte is present. For substances with an MRL, the value of *α* is considered to be 5%. The calculation was performed by analysing 20 blank samples fortified with the analyte at the MRL level. The concentration of the MRL plus 1.64 times the standard deviation corresponds to the CC*α* (*α* = 5%). The detection capability (CC*β*) is the lowest amount of the substance that can be detected, identified, and/or quantified in a sample with an acceptable error probability (*β*). For substances with an MRL, the determination of CC*β* can be accomplished by the analysis of 20 blank samples fortified with the analyte in the decision limit (CC*α*). The value of CC*α* plus 1, 64 times the standard deviation, corresponds to the CC*β* (*β* = 5%).

For each sulfonamide and trimethoprim, the LOD and LOQ were established by analysing the fortified matrix with standard solution of the analytes. LOD was determined based on signal-to-noise approach. Thus, LOD was expressed as the lowest concentration with a signal equal to three times the signal-to-noise ratio. The LOQ was taken as the first level of the analytical curve, which was measured with acceptable precision (CV ≤ 20%) [26].

## 3. Results and Discussion

The representative sulfonamide veterinary drugs were chosen based on a study of their use in fish farming around the world, those monitored by the Brazilian National Plan for Control of Residues and Contaminants (PNCRC/Fish) of the Brazilian Ministry of Agriculture, Livestock, and Food Supply and those used for other animal species that could potentially be illegally employed in fish farming. Thus, sulfamethazine, sulfathiazole, sulfadimethoxine, sulfamerazine, sulfamethoxazole (monitored by the PNCRC/Fish [8]), sulfapyridine, sulfamethoxypyridazine, and trimethoprim (regulated for veterinary use [7], although not regulated for use in fish farming in Brazil) were selected. The maximum residue limit (MRL) adopted for all the sulfonamides (individual or the combined total residues) was 100 *μ*g·kg^−1^, and 50 *μ*g·kg^−1^ for trimethoprim [7].

### 3.1. Sample Preparation Based on QuEChERS

Dispersive solid-phase extraction (d-SPE) technique and QuEChERS have been previously used for the determination of veterinary drug residues in animal fluids and tissues [16, 27, 28], but not for the concomitant determination of sulfonamides and trimethoprim in fish fillet. It is well known that the step of sample preparation (extraction of analytes and cleanup of the extract) is crucial. This approach can influence the magnitude of the matrix effect, depending on the amount of endogenous substances from which it is coextracted. Acetonitrile has been widely used in the extraction of analytes from complex matrices as it extracts analytes with few interfering compounds (e.g., low amount of lipophilic coextractives from the sample) and further promotes the precipitation of proteins. This is necessary because the lower the quantity of interfering content present in the extract, the less matrix effect is observed, which leads to a better quality analysis [29].

Kruve et al. [30] reported the minimizing matrix effect in LC-ESI-MS analysis by using extrapolative dilution. It was demonstrated by several tests using QuEChERS sample preparation procedure that the use of a greater volume of acetonitrile for analyte extraction of complex matrices tends to reduce the matrix effect, possibly eliminating the matrix effect if a suitable dilution is achieved. It should be mentioned that although LC-ESI-QTOF/MS technique is very selective, possible interference caused by matrix substances can lead to suppression or an increase in the ionization of the analytes of interest [31]. Thus, this study explores the extraction of sulfonamides and trimethoprim by using QuEChERS procedure making use of acetonitrile as the extracting solvent and extrapolative dilution.

Preliminary studies have shown that for the quantification of sulfonamides and trimethoprim in tilapia fillet using the proportion of acetonitrile : sample 4 : 1 (v/w) showed the best results with fewer coextracts, thus decreasing the presence of interfering compounds. It is noteworthy that although the amount of sample used in this study was four times lower than that used by Lehotay et al. [21], it was possible to achieve an LOQ of 5 ng·g^−1^ for all analytes, consequently to the LC-ESI-QTOF/MS system used. Literature data show that the LOQ for SDZ was 36 ng·g^−1^, using 5 g of the sample [32]. Stubbings and Bigwood [33] showed an LOQ for SP, STZ, SMZ, SDMX, SMX, and SMR of 50 ng·g^−1^, also using 5 g of the sample.

The addition of salts to promote the salting out effect has been shown to enhance the optimization of the analyte recovery percentages in multiresidue methods since it increases the solubility of these molecules in the organic phase [34, 35]. In the QuEChERS approach reported by Lehotay et al. [21], 6 g of anhydrous magnesium sulfate and 2.5 g of sodium acetate trihydrate were used. In the present method for extracting sulfonamides and trimethoprim from tilapia fillet, 2 g of anhydrous magnesium sulfate and 0.75 g of sodium acetate trihydrate were employed. At the cleanup step, PSA and/or C18 were used. Since no significant variation was observed between them in relation to recovery values, we opted for the use of PSA only. This finding may be observed because the fat content in tilapia fillet is low. There are studies in matrices that have considerably higher fat content in which the concomitant use of PSA and C18 is required for a better cleanup of the sample extract [33].

### 3.2. Identity Confirmation of Analytes

On the basis of Commission Decision 2002/657/EC [22], the identity confirmation of a substance is performed by a system of identification points (IPs). The mass accuracy of a high-resolution mass spectrometer acquires 2 IPs for the precursor ion and 2.5 for each transition product. The resolution of mass spectrometer used in this study (SYNAPT HDMS Q-TOF) is more than 10,000, which fall within the criteria established by the guide as a high-resolution MS. Under the conditions selected, the protonated molecule and one fragmented ion for each analyte could be monitored, thus reaching the requirements to confirm their identity in accordance with Commission Decision 2002/657/EC [22]. For the quantitative purpose, only the sulfonamides and trimethoprim molecular ions were monitored.

### 3.3. Analytical Method Validation

The method selectivity was evaluated by analysing ten samples free of analytes (blank samples) and comparing them to the chromatograms obtained from samples spiked with the sulfonamides and trimethoprim. Peaks for interfering compounds with the same retention times as the analytes of interest with the same *m/z* were not observed. Therefore, the method performed is satisfactorily selective.

**Figure 3:**
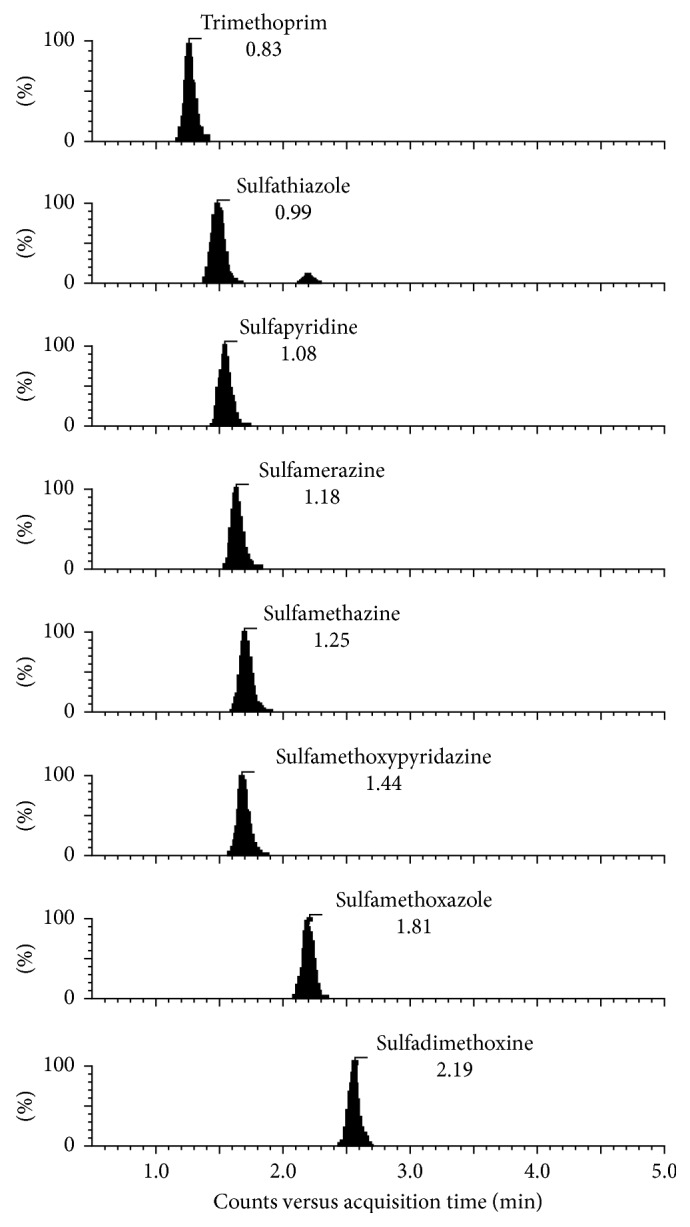
Extracted chromatograms of spiked samples with sulfonamides and trimethoprim at concentration of 50.0 ng·g^−1^.

Figure 3 shows the chromatograms of each analyte studied.

To study the linearity, sensitivity, and matrix effect, the analytical results at the following concentrations were compared: 5.0, 12.5, 25.0, 50.0, 75.0, 100.0, 125.0, and 250.0 ng·mL^−1^. Measurements were carried out for the analytes dissolved in the solvent, in the fortified extract, and in the fortified blank matrix (matrix-matched). The matrix effect, expressed as a percentage, was calculated from the division between the areas obtained for the analyte in solvent and in the fortified extract, at the same concentration level [36]. The highest matrix effect value observed was 18,98%, which is below the maximum acceptable value by the validation guides (20%) [22]. Thus, the matrix effect was considered irrelevant for this method. However, when comparing the analytical curves in extract with the curves in the fortified blank matrix, it was noted that the slope (angular coefficient) of the curve for the matrix-matched sample was much lower, indicating the loss of analytes (sulfonamides and trimethoprim) during sample preparation step (extraction and cleanup). Thus, for the present method a matrix-matched analytical curve must be employed.

Accuracy was evaluated from recovery tests (%), as recommended by the Commission Decision 2002/657/EC when no certified reference material (CRM) is available [22]. The experiment was carried out through the recovery test of the spiked samples at 3 levels (10.0, 20.0, and 40.0 ng·g^−1^), evaluating each level using 5 independent replicates on 3 consecutive days. Analytes SP, SMR, and TMP had satisfactory recovery values (between 79.5 and 103.6%), SMZ and SMPD showed intermediate recoveries (between 64.6 and 80.0), and STZ, SDMX and SMX exhibit lower recovery values (between 38.4 and 52.9) (Table 2). Low recovery values for sulfonamides have been reported. Won et al. [37] reached a recovery of 58.8% for SDMX after extraction of this molecule from marine products, such as common eel, blue crab, shrimp, and flatfish, among others. Sulfonamides' low recoveries have also been reported in other matrices. Summa et al. [38] report recoveries for SMX and SDM, extracted from eggs, around 60% and 55%, respectively. A review dealing about the presence of sulfonamides in edible tissues reports recoveries of various sulfonamides ranging from 40 to 67% for honey, 45–85% for pork veal, and 57–63% for salmon muscle [39]. Although recovery values found for some sulfonamides were below the percentage established in the validation guide [22], the method has been shown to be precise (CV% found is within the value specified in the validation guide), and the required LOQ was achieved, which leads us to consider that the method is suitable for the intended purpose. Nevertheless, this corroborates the need to use matrix-matched analytical curves for the quantification of the analytes in samples of unknown origin.

The precision of the method was determined through intraday precision (repeatability) and interdays (intermediate precision) at three spiked levels and was expressed as coefficient of variation (CV%). The intraday and interdays precision were evaluated in the concentration levels at 10.0, 20.0, and 40.0 ng·g^−1^, with 5 replicates at each level. Working in this concentration range, we can ensure the precision and accuracy since in the most dispersive points, the CV is ≤20%. The repeatability (analysed on the same day and same equipment) and the interdays precision (intermediate precision) are shown in Table 3.

For compounds with concentration levels lower than 100 ng·g^−1^, the Commission Decision 2002/657/EC [22] and Brazilian validation guideline [23] recommend a maximum acceptable CV ≤ 20%. As shown in Table 3, the validation parameters (intraday and interdays precision) meet the specifications recommended by both guides since they recommend a CV ≤ 20%.

The decision limit (CC*α*) is a parameter that takes into account the precision of the method for establishing a critical reference level, from which we can conclude that a sample is classified as nonconforming with a probability of error of 5%. An additional critical parameter, detection capability (CC*β*), is calculated for use with nonconforming samples in order to confirm their concentration, and their identities are confirmed with an error probability of 5% (*β* = 5%).

The decision limit (CC*α*) and detection capability (CC*β*) values for each of the analyte studied are shown in Table 4. For sulfonamides, values varied from 102.6 to 120.0 *µ*g·kg^−1^ and from 111.7 to 140.1 *µ*g·kg^−1^ for CC*α* and CC*β*, respectively. For trimethoprim, those values were 70.0 and 89.9 *µ*g·kg^−1^, respectively. Thus, considering the MRL values of 100 *µ*g·kg^−1^ and 50 *µ*g·kg^−1^, respectively, for sulfonamides and trimethoprim, established by the regulatory framework of the European Union in fin fish [7], we can conclude that the method reported here is suitable for application in surveillance programmes of residues of sulfonamides and trimethoprim in fin fish muscle samples.

The evaluation of LOD and LOQ for the determination of sulfonamides and trimethoprim residues in tilapia fillet was performed using the matrix-matched analytical curve fortified with the analytes. The LOD and LOQ of the method were 1.0 ng·g^−1^ and 5.0 ng·g^−1^ for all sulfonamides and trimethoprim, respectively. The LOQ was validated by analyse of 10 replicates that showed a CV ≤ 20% for all of the analytes. This indicates that due to the low value of LOQ obtained, the method can be used by restrictive regulatory agencies of countries such as Japan [40], which, for the multiresidue method intended for quantification of veterinary drug residues in animal and fishery products, adopt for individual sulfonamides and trimethoprim a LOQ value of 10 ng·g^−1^ and 20 ng·g^−1^, respectively.

### 3.4. Analysis of Incurred Samples

To assess the method developed, analysis was performed on genuinely contaminated (incurred) fish samples obtained from an experiment in laboratory where the fishes were exposed to SMZ through the feed. This study was related to the effects of dietary exposure to SMZ on the haematological parameters and hepatic oxidative stress biomarkers in Nile tilapia [25]. The residue of SMZ in the muscle of 10 independent samples analysed in the same day was 1,062.9 ± 53.2 ng·g^−1^ (mean value ± standard deviation), and the precision (CV%) was 5.0%. Due to the high concentration levels, the extract of the samples was diluted prior to injection to adjust the concentration to fit the range of the analytical curve. This corroborates the precision of the method and provides confidence that it is appropriate for the intended purpose and can be used by regulatory agencies in health surveillance programs, as well as in pharmacokinetics and residue depletion studies.

## 4. Conclusions

A multiresidue method for determination of sulfonamides STZ, SMX, SMR, SMPD, SDMX, SP, and SMZ and trimethoprim (TMP) in tilapia fillet was developed and validated. The analytes selected were those most frequently used worldwide in fish farming and those with the greatest potential for illegal use. QuEChERS approach with extrapolative dilution was shown to be a simple and inexpensive sample preparation process that can be easily used in routine analysis. Quantitation by liquid chromatography-quadrupole time-of-flight mass spectrometry (LC-ESI-QTOF/MS) showed to be a selective and low detectability method. Thus, the method is suitable for application in monitoring programmes of residues of sulfonamides and trimethoprim in tilapia fillet, even by countries such as Japan that adopt low LOQ values for analytical methods to be used in food for determination of residues of substances such as veterinary drugs. Also, it was shown to be appropriate to be used in pharmacokinetic and residue depletion studies.

## Figures and Tables

**Table 1:** Elemental composition, retention time, the *m/z* experimental (precursors and fragment) ions, and mass error determined in standard solution for the studied analytes.

Compound	Molecular formula	Retention time (min)	Monoisotopic mass (Da)	*m/z* experimental [M + H]^+^ (Da)	Mass error (ppm)	*m/z* experimental fragment ion (Da)
Trimethoprim	C_14_H_18_N_4_O_3_	0.83	290.1379	291.1460	1.0	123.0592
Sulfapyridine	C_11_H_11_N_3_O_2_S	1.08	249.0572	250.0650	0.0	156.0128
Sulfamerazine	C_11_H_12_N_4_O_2_S	1.18	264.0681	265.0760	0.4	108.0483
Sulfathiazole	C_9_H_9_N_3_O_2_S_2_	0.99	255.0136	256.0210	1.6	156.0127
Sulfamethazine	C_12_H_14_N_4_O_2_S	1.25	278.0837	279.0920	1.4	108.0475
Sulfadimethoxine	C_12_H_14_N_4_O_4_S	2.19	310.0736	311.0810	1.3	156.0771
Sulfamethoxazole	C_10_H_11_N_3_O_3_S	1.81	253.0521	254.0600	0.4	156.0124
Sulfamethoxypyridazine	C_11_H_12_N_4_O_3_S	1.44	280.0630	281.0710	0.7	156.0125

**Table 2:** Validation parameters of sulfonamides and trimethoprim.

Validation parameters	Sulfonamides and trimethoprim							
	SP	STZ	SMZ	SDMX	SMX	SMPD	SMR	TMP
Working range (ng·g^−1^)	5–250	5–250	5–250	5–250	5–250	5–250	5–250	5–250
Linearity (*R*^2^)	0.9958	0.9914	0.9922	0.9992	0.9994	0.9935	0.9964	0.9984
Sensibility	1174.07	633.811	2324.3	2670.56	1626.52	2057.08	1345.72	1588.92
Matrix effect (%)								
12.5 ng·g^−1^	−18.98	−2.07	−11.19	−3.58	−5.22	9.57	−3.68	9.65
50 ng·g^−1^	1.60	14.71	0.37	−3.28	1.13	−4.15	1.85	−0.12
100 ng·g^−1^	0.13	−7.02	0.46	1.61	−1.77	3.74	0.79	−0.03
Accuracy (% recovery (CV%))								
10 ng·g^−1^	83.9 (14.4)	52.9 (19.2)	69.1 (19.4)	49.6 (17.8)	45.4 (11.9)	80.0 (9.4)	79.5 (15.4)	92.0 (13.2)
20 ng·g^−1^	91.5 (19.3)	38.4 (17.2)	72.4 (11.8)	47.4 (3.4)	41.8 (4.2)	66.8 (9.0)	85.4 (16.7)	88.2 (17.7)
40 ng·g^−1^	103.6 (19.0)	41.3 (18.6)	68.0 (19.1)	51.1 (4.5)	43.5 (10.0)	64.6 (5.5)	81.2 (15.0)	91.5 (16.2)
LOD (ng·g^−1^)	1.0	1.0	1.0	1.0	1.0	1.0	1.0	1.0
LOQ (ng·g^−1^)	5.0	5.0	5.0	5.0	5.0	5.0	5.0	5.0

SP, sulfapyridine; STZ, sulfathiazol; SMZ, sulfamethazine; SDMX, sulfadimethoxine; SMX,  sulfamethoxazole; SMPD,  sulfamethoxypyridazine; SMR, sulfamerazine; TMP, trimethoprim; LOD, limit of detection; LOQ,  limit of quantitation; CV, coefficient of variation.

**Table 3:** Intraday and interdays precision of sulfonamides and trimethoprim.

Validation parameters	Sulfonamides and trimethoprim							
	SP	STZ	SMZ	SDMX	SMX	SMPD	SMR	TMP
Intraday precision (CV%)								
10 ng·g^−1^	12.8	11.7	11.6	7.7	6.4	7.1	11.3	8.3
20 ng·g^−1^	11.2	8.2	8.3	7.7	5.9	13.9	13.6	7.8
40 ng·g^−1^	12.9	14.9	15.0	12.3	7.9	6.1	14.5	10.6
Interdays precision (CV%)								
10 ng·g^−1^	14.4	19.2	19.4	17.8	11.9	9.4	15.4	13.2
20 ng·g^−1^	19.3	17.2	11.8	3.4	4.2	9.0	16.7	17.7
40 ng·g^−1^	19.0	18.6	19.1	4.5	10.0	5.5	15.0	16.2

SP, sulfapyridine; STZ, sulfathiazol; SMZ, sulfamethazine; SDMX, sulfadimethoxine; SMX, sulfamethoxazole; SMPD, sulfamethoxypyridazine; SMR, sulfamerazine; TMP, trimethoprim; CV, coefficient of variation.

**Table 4:** CC*α* and CC*β* values for sulfonamides and trimethoprim in tilapia fillet.

Validation parameters	Sulfonamides^a^ and trimethoprim^b^							
	SP	STZ	SMZ	SDMX	SMX	SMPD	SMR	TMP
Limit of decision (CC*α*), ng·g^−1^	119.8	110.9	114.0	102.6	102.9	105.9	120.0	70.0
Detection capability (CC*β*), ng·g^−1^	139.7	121.7	122.0	117.1	111.7	118.2	140.1	89.9

SP, sulfapyridine; STZ, sulfathiazole; SMZ, sulfamethazine; SDMX, sulfadimethoxine; SMX, sulfamethoxazole; SMPD, sulfamethoxypyridazine; SMR, sulfamerazine; TMP, trimethoprim. ^a^The MRL value adopted for the calculation of CC*α* and CC*β* for all sulfonamides was 100 ng·g^−1^ [6]. ^b^The MRL value adopted for the calculation of CC*α* and CC*β* for TMP was 50 ng·g^−1^ [6].
